# In Vitro Radiobiological Evaluation of [^64^Cu]CuCl_2_ for Theranostic Applications

**DOI:** 10.3390/ph19071033

**Published:** 2026-07-02

**Authors:** Francesca Porto, Silvia Pasquini, Chiara Contri, Martina Cappello, Giorgia Speltri, Alessandra Boschi, Licia Uccelli, Rebecca Napolitano, Lorenza Marvelli, Katia Varani, Giovanni Di Domenico, Petra Martini, Fabrizio Vincenzi

**Affiliations:** 1 Department of Translational Medicine, University of Ferrara, Via L. Borsari, 46, 44121 Ferrara, Italy; francesca.porto@unife.it (F.P.); silvia.pasquini@unife.it (S.P.); chiara.contri@unife.it (C.C.); martina.cappello@unife.it (M.C.); rebecca.napolitano@unife.it (R.N.); katia.varani@unife.it (K.V.); fabrizio.vincenzi@unife.it (F.V.); 2 Department of Chemical, Pharmaceutical and Agricultural Sciences, University of Ferrara, Via L. Borsari, 46, 44121 Ferrara, Italy; alessandra.boschi@unife.it (A.B.); lorenza.marvelli@unife.it (L.M.); 3 Department of Physics and Earth Sciences, University of Ferrara, Via Saragat, 1, 44122 Ferrara, Italy; giovanni.di.domenico@unife.it; 4 Department of Environmental and Prevention Sciences, University of Ferrara, Via L. Borsari, 46, 44121 Ferrara, Italy; petra.martini@unife.it

**Keywords:** [^64^Cu]CuCl_2_, peritoneal carcinomatosis, theranostic agent, cell dosimetry

## Abstract

**Background/Objectives**: Intrinsic genetic instability and the marked heterogeneity of malignant cell populations represent significant clinical challenges in oncology, often limiting the efficacy of conventional receptor-targeted and antigen-based therapies. To overcome these limitations, [^64^Cu]CuCl_2_ has emerged as a particularly promising theranostic agent because it combines PET imaging (β^+^ emission) with therapeutic effects (β^−^ particles and Auger electrons). In particular, Auger electrons, when delivered to the cell nucleus, induce severe DNA damage due to their high linear energy transfer and very short tissue range. This work aimed to deepen existing preclinical knowledge by providing a comprehensive in vitro analysis of the interactions of [^64^Cu]CuCl_2_ with various human cancer cell lines—specifically, the breast adenocarcinoma (MDAf-MB-231) and gastric carcinoma (NCI-N87) cell lines—and a healthy control (IMR-90 normal human fetal lung fibroblasts). **Methods**: We focused on evaluating cellular uptake, subcellular localization, impact on metabolic activity, and induction of apoptosis. Cell lines (MDA-MB-231, NCI-N87, IMR-90) were exposed to increasing activities of [^64^Cu]CuCl_2_ (10, 100, and 250 µCi/mL). Uptake was assessed in both nuclear and cytoplasmic compartments after 4 h. Metabolic activity and apoptosis/necrosis were evaluated at 96 and 120 h post-treatment. **Results**: Tumor cell lines demonstrated significantly higher [^64^Cu]CuCl_2_ uptake, particularly at the nuclear level, compared to healthy controls. A marked decrease in metabolic activity and an increase in apoptosis were observed in MDA-MB-231 and NCI-N87 cells (from 50% to 90% and 5% to 60% apoptosis, respectively). In contrast, IMR-90 cells exhibited minimal cytotoxic response (≤20%), suggesting a preferential response in the malignant cell models tested. **Conclusions**: [^64^Cu]CuCl_2_ induced distinct patterns of intracellular accumulation and biological response among the investigated cell models, with cancer cells displaying greater nuclear uptake and apoptotic susceptibility than non-malignant cells. These findings provide a high-resolution radiobiological baseline and microdosimetric validation, supporting the rigorous design of future, dedicated in vivo preclinical investigations to evaluate the translational potential of ionic [^64^Cu]CuCl_2_.

## 1. Introduction

Intrinsic genetic instability and the marked heterogeneity of malignant cell populations represent significant clinical challenges in oncology, often limiting the efficacy of conventional receptor-targeted and antigen-based therapies.

Peritoneal carcinomatosis (PC) represents a paradigmatic example of this condition. PC is an advanced stage of neoplastic disease in which malignant tumor cells of gynecological or gastrointestinal origin metastasize in the peritoneal cavity. In advanced cases of gastric cancer (GC), one of the main causes of cancer-related deaths worldwide, metastatic dissemination to the peritoneum is the main form of recurrence after surgical resection. Peritoneal metastasis (PM) of GC, the corresponding clinical condition of PC, is associated with extremely poor prognosis, with only 3–4 months of median survival time [1]. Aiming to prolong the survival of patients with PM, the most frequent treatment applied consists of combined multimodal methods (e.g., complete cytoreductive surgery combined with hyperthermic intraperitoneal chemotherapy and systemic chemotherapy). The need for innovative therapeutic strategies for treating PM is recognized by the medical and scientific community. Beyond standard methods, new treatment strategies for PM are under investigation.

Some PM cells have been found to express the human epidermal growth factor receptor 2 (HER2) thus prompting preclinical studies based on radioimmunotherapy (RIT), a targeted radioisotope treatment using an antibody as a carrier of therapeutic radioisotopes, like the RIT study on PMGC mouse model with astatine-211 (^211^At-trastuzumab) [2]. However, this strategy is limited to the treatment of cancer cells expressing the specific receptor involved in the targeted therapy. Indeed, one of the most formidable problems that makes the search for an effective cancer therapy so difficult arises from the marked genetic instability of cancerous cells, which leads to the growth of different populations of malignant cells within the same tumor lesion. This inherent heterogeneity is reflected in the dynamic behavior of the cancerous cell, which can continuously modify its biomolecular profile by adapting to environmental conditions.

Copper-64 chloride ([^64^Cu]CuCl_2_) has emerged as a particularly promising theranostic agent, offering a dual capability for both diagnostic imaging and therapeutic intervention. Its unique radiophysical properties, which include decay via positron emission (β^+^) for PET (Positron Emission Tomography) imaging and negative beta particles (β^−^) and Auger electrons for therapeutic cytotoxicity, make it a versatile radionuclide. The therapeutic efficacy of [^64^Cu]CuCl_2_ is intrinsically linked to its decay characteristics: while β^−^ particles have sufficient tissue penetration to affect hundreds of tumor cells, Auger electrons, characterized by their low energy (<5 keV) but high linear energy transfer (LET) (4–26 keV/μm) and very limited tissue penetration depth (<1 μm), are particularly potent when precisely targeted to the cell nucleus. This precise subcellular targeting is crucial, as the highest lethal effect is achieved when radiation is emitted in close proximity to the cell nucleus, causing irreversible DNA damage [3,4].

The biological rationale for using [^64^Cu]CuCl_2_ stems from the altered copper metabolism in tumor cells, often referred to as “cuproplasia” or copper-regulated cell proliferation [5]. Copper is an essential metabolic cofactor and a dynamic signaling metal, with tumor cells exhibiting an increased requirement for this element to support rapid growth and metastasis [6]. The selective uptake of Cu^2+^ ions by tumor cells is mediated by transporters such as hCTR1 (human copper transporter-1). Specifically, the activated protein ATOX1 plays a crucial role in transporting copper ions to the nucleus, where they contribute to cell proliferation. This mechanism explains the observation that highly proliferating cancerous tissues typically have higher copper levels compared to normal tissues [7].

Among clinically investigated Auger electron emitters, ^64^Cu is distinctive because it can be administered in its free ionic form ([^64^Cu]CuCl_2_), allowing its biodistribution to be governed by endogenous copper metabolism rather than by an exogenous targeting vector, thereby offering the potential to localize in close proximity to DNA and induce Auger electron-mediated damage [8,9].

Based on the encouraging results obtained in clinical trials designed to evaluate the efficacy of the [^64^Cu]Cu^2+^ ion against glioblastoma, the European Medicines Agency (EMA) granted orphan drug status in 2023 to [^64^Cu]CuCl_2_, administered intravenously as an aqueous buffered solution, for the treatment of glioblastoma; more recently, the U.S. Food and Drug Administration (FDA) has also granted Orphan Drug Designation for the same indication [10]. Experiments on glioblastoma therapy with ionic ^64^Cu show that nuclear localization occurs only in abnormal cells, not in healthy ones.

Recent advancements in 3D culture models, such as glioblastoma spheroids (including U87, U373, and T98G lines), have significantly expanded our understanding of [^64^Cu]CuCl_2_ beyond initial clinical observations [11]. These findings support the hypothesis that nuclear translocation occurs preferentially in malignant cells, although the degree of sensitivity can vary based on the tumor’s genetic profile, such as TP53 status. Due to the limited number of studies on this topic, the scientific community emphasizes the need for further experimental tests to gain a comprehensive understanding of ^64^Cu ion behavior in tumor cells and determine its potential for cancer treatment [12].

Unlike receptor-targeted approaches, whose efficacy depends on the presence and stability of specific molecular targets, the therapeutic rationale of ionic [^64^Cu]CuCl_2_ relies on the altered copper metabolism frequently observed in malignant cells. Therefore, evaluating biologically distinct tumor models is essential to determine whether the uptake and radiobiological effects of ionic copper are confined to specific cancer types or can be observed across heterogeneous tumor phenotypes.

To explore whether the biological effects of ^64^Cu are maintained across tumor contexts with distinct phenotypic and metabolic features, we selected two human carcinoma cell lines with markedly different biological profiles: NCI-N87 and MDA-MB-231. NCI-N87 is a standard HER2-overexpressing gastric carcinoma model with a clear epithelial morphology, representative of a clinically relevant subtype of gastric cancer and relevant to peritoneal dissemination. MDA-MB-231, on the other hand, is a highly aggressive, triple-negative breast cancer model known for its invasive, mesenchymal-like traits and phenotypic plasticity. Peritoneal metastases from breast cancer are also a rare and challenging clinical presentation [13]. Because copper uptake and intracellular handling are strongly influenced by CTR1 and by the metabolic state of tumor cells, comparing these two distinct models may help clarify whether differences in ^64^Cu response reflect intrinsic biological features rather than tissue of origin alone. Therefore, MDA-MB-231 was selected not only as a model of peritoneal carcinomatosis, but also as a complementary model of an aggressive and biologically distinct carcinoma, allowing us to assess whether the effects of ionic ^64^Cu are preserved across tumors with different phenotypic characteristics.

This work aims to deepen existing preclinical knowledge by providing a comprehensive analysis of the interactions of [^64^Cu]CuCl_2_ with various human cancer cell lines and a healthy control, focusing on cellular uptake, subcellular localization, impact on metabolic activity, and induction of apoptosis. By integrating these detailed in vitro observations, we seek to enhance the understanding of the cellular radiobiological behavior of [^64^Cu]CuCl_2_, address observed differences in cellular responses, and to define model-dependent differences in uptake, metabolic activity, apoptosis, and dosimetric relationships.

## 2. Results

### 2.1. [^64^Cu]CuCl_2_ Uptake in Cellular Compartments

The cellular uptake and subcellular distribution of [^64^Cu]CuCl_2_ were assessed in the breast cancer cell line MDA-MB-231 and the gastric carcinoma cell line NCI-N87, with the IMR-90 fibroblast cell line used as a control.

**Figure 1:**
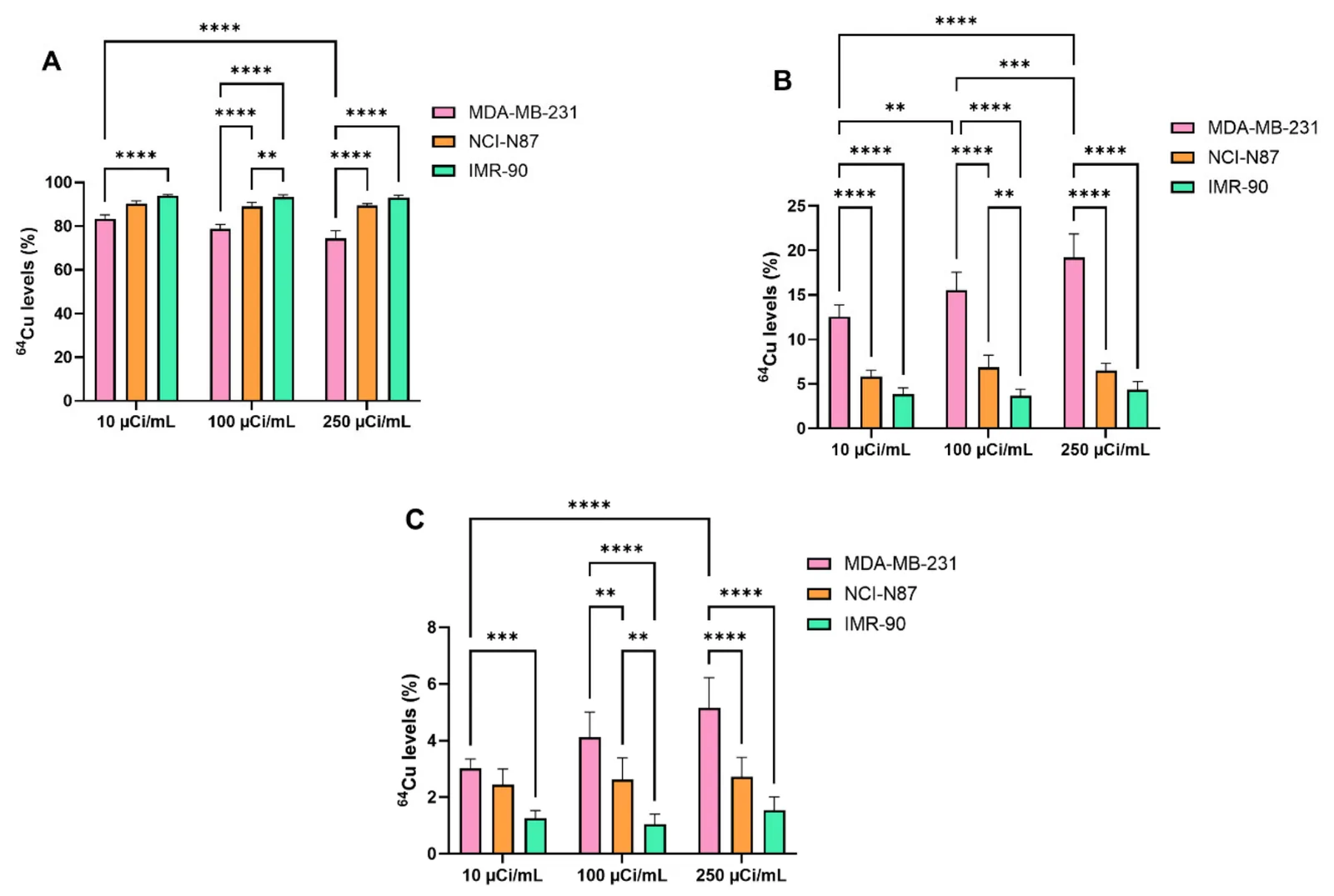
Cellular uptake and compartmental distribution of [^64^Cu]CuCl_2_**.** Uptake and cellular distribution of [^64^Cu]CuCl_2_ in the cell culture supernatant (**A**), cytoplasm (**B**), and nucleus (**C**) after 4-h incubation with MDA-MB-231, NCI-N87, and IMR-90 cell lines. Cell lysis and extraction of separate cytoplasmic and nuclear protein fractions were performed using NE-PER™ Nuclear and Cytoplasmic Extraction Reagents (Thermo Fisher Scientific, Waltham, MA, USA). The single fraction radioactivity was then measured with a liquid scintillation counter. Data are shown as the mean ± SEM of five independent experiments and were analyzed by two-way ANOVA with Tukey’s multiple comparison test. ** *p* < 0.01; *** *p* < 0.001; **** *p* < 0.0001.

Radioactivity distribution among the supernatant, cytoplasmic, and nuclear fractions was assessed after a 4-h incubation with [^64^Cu]CuCl_2_ at 10, 100, or 250 µCi/mL. As shown in Figure 1A, the majority of ^64^Cu radioactivity remained in the supernatant, as expected. Notably, MDA-MB-231 cells exhibited lower supernatant radioactivity, suggestive of greater ^64^Cu intracellular uptake compared to the other cell lines. This reduction was statistically significant across all tested concentrations when compared with IMR-90, and at 100 µCi/mL and 250 µCi/mL when compared with NCI-N87 (Figure 1A). The percentage of ^64^Cu remaining in the supernatant was significantly lower after incubation of MDA-MB-231 cells with 250 μCi/mL compared to 10 μCi/mL, suggesting a dose-dependent uptake in these cells (Figure 1A). In the gastric carcinoma cell line NCI-N87, only the 100 µCi/mL concentration resulted in significantly lower supernatant ^64^Cu levels compared to IMR-90 (Figure 1A). IMR-90 cells consistently showed the highest levels of ^64^Cu retention in the supernatant, suggesting limited uptake (Figure 1A).

^64^Cu cytoplasmic uptake in MDA-MB-231 cells followed a dose-dependent pattern and was significantly higher than in the other two cell lines, consistent with the lower levels observed in the supernatant (Figure 1B). In NCI-N87 cells, only the 100 μCi/mL concentration induced a significantly higher cytoplasmic uptake compared to IMR-90 (Figure 1B).

Regarding nuclear uptake, MDA-MB-231 cells exhibited a significantly higher accumulation of ^64^Cu at 250 µCi/mL of [^64^Cu]CuCl_2_ compared to 10 µCi/mL, indicating a dose-dependent nuclear localization (Figure 1C). Moreover, ^64^Cu nuclear uptake in MDA-MB-231 was significantly higher compared to IMR-90 across all tested concentrations and compared to NCI-N87 at 100 µCi/mL and 250 µCi/mL. In NCI-N87 cells, incubation with 100 µCi/mL of [^64^Cu]CuCl_2_ induced significantly greater ^64^Cu nuclear accumulation compared to IMR-90. No dose-dependent differences in nuclear uptake were observed in the IMR-90 cells (Figure 1C).

These findings highlight a differential intracellular distribution of ^64^Cu, with cancer cells, particularly MDA-MB-231, showing enhanced internalization in the cytoplasmic and nuclear compartments.

### 2.2. Induction of Apoptosis by [^64^Cu]CuCl_2_ Treatment

**Figure 2:**
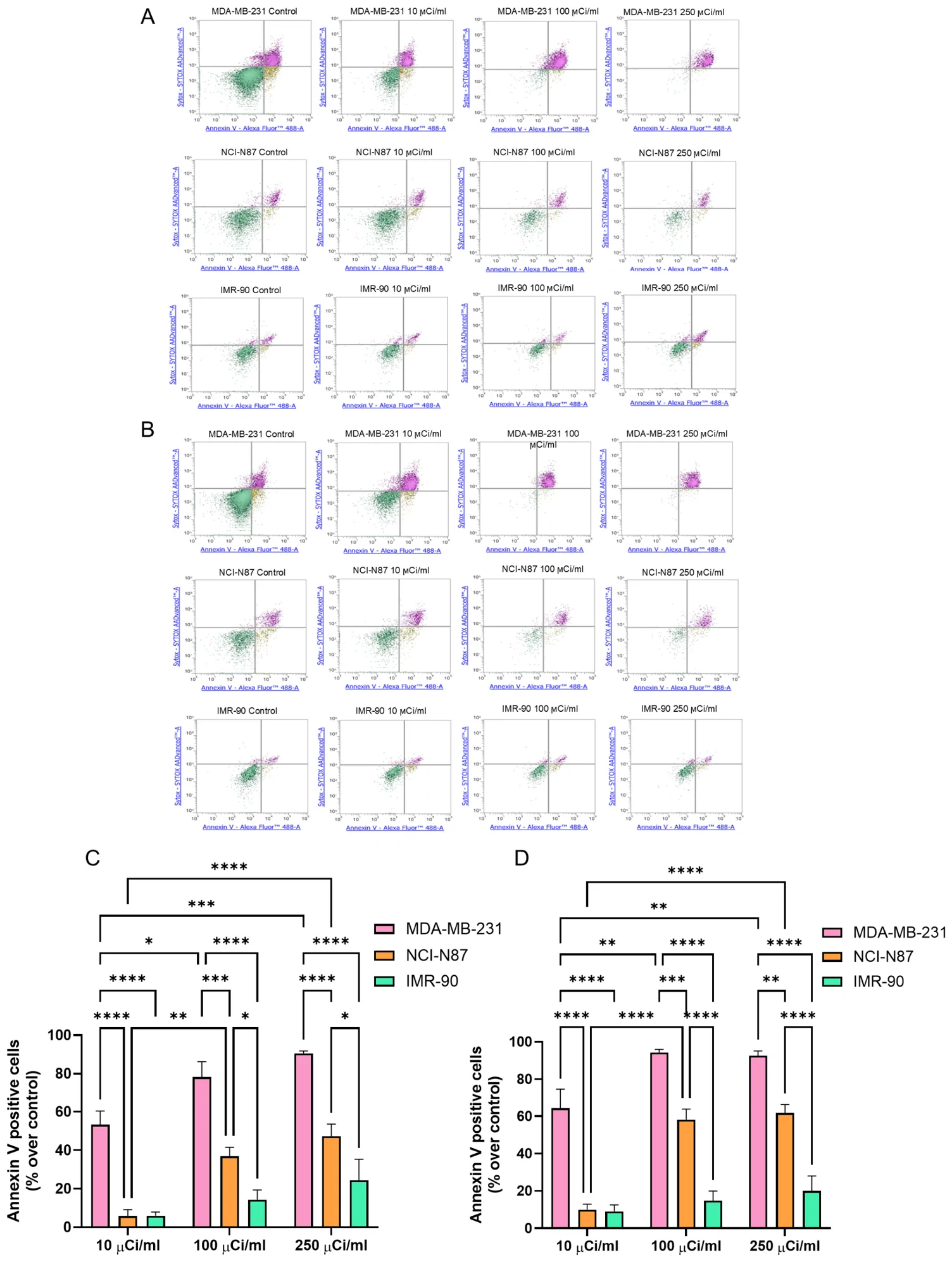
Flow cytometry analysis. Representative density plots of flow cytometry analysis of MDA-MB-231, NCI-N87, and IMR-90 cells exposed to different concentrations of [^64^Cu]CuCl_2_ for 96 h (**A**) and 120 h (**B**). Cells were double-stained with Annexin V Alexa Fluor™ 488 Ready Flow Conjugate and SYTOX™ AADvanced™ Dead Cell Stain (Invitrogen™, Thermo Fisher Scientific, Waltham, MA, USA). Annexin V-negative/SYTOX-negative cells (bottom-left quadrant) represent living cells; Annexin V-negative/SYTOX-positive cells (top-left quadrant) represent necrotic cells; Annexin V-positive/SYTOX-negative cells (bottom-right quadrant) represent early apoptotic cells; Annexin V-positive/SYTOX-positive cells (top-right quadrant) represent late apoptotic cells. Histogram showing the percentage of Annexin V-positive cells after 96 h (**C**) and 120 h (**D**) of treatment with [^64^Cu]CuCl_2_ in the three cell lines used. The percentage of apoptotic cells was calculated by subtracting the percentage of Annexin V-positive cells in the control condition from each treated sample. Data are presented as the increase in Annexin V-positive cells (% over control). Data are expressed as the mean ± SEM of five independent experiments and were analyzed by two-way ANOVA with Tukey’s multiple comparison test. * *p* < 0.05; ** *p* < 0.01; *** *p* < 0.001; **** *p* < 0.0001.

Apoptotic cell death was quantified by flow cytometry in the same panel of cell lines following exposure to increasing concentrations of [^64^Cu]CuCl_2_ for 96 and 120 h (Figure 2A,B). After 96 h of treatment with 100 µCi/mL and 250 µCi/mL concentrations, a significant increase in apoptosis was observed in MDA-MB-231 and NCI-N87 tumor cells compared to healthy control cells (Figure 2C). MDA-MB-231 cells exhibited the strongest response, with greater induction of apoptosis not only compared to IMR-90 control cells but also to NCI-N87 tumor cells, across all tested concentrations of [^64^Cu]CuCl_2_.

At 120 h, apoptosis levels increased further, with MDA-MB-231 cells continuing to show the most robust response. Exposure to 10 µCi/mL [^64^Cu]CuCl_2_ resulted in a 64% increase in apoptosis above control levels in MDA-MB-231, while NCI-N87 and IMR-90 showed 10% and 9%, respectively (Figure 2D). At 100 µCi/mL, apoptosis reached near-total levels in MDA-MB-231 and was 58% above controls in NCI-N87, both showing statistically significant differences compared to IMR-90, which only reached 15% (Figure 2D). In tumor cells, but not in control cells, apoptosis levels were significantly higher after exposure to 100 µCi/mL than to 10 µCi/mL, indicating a dose-related effect. However, exposing cells to 250 µCi/mL for both 96 and 120 h did not significantly change the percentage of apoptosis with respect to 100 µCi/mL, suggesting that 100 µCi/mL is sufficient to achieve a maximal effect (Figure 2C,D).

Collectively, these findings indicate that [^64^Cu]CuCl_2_ preferentially induces apoptosis in malignant cells, with minimal effects on healthy control cells.

### 2.3. Effects of [^64^Cu]CuCl_2_ Treatment on XTT-Derived Metabolic Activity

**Figure 3:**
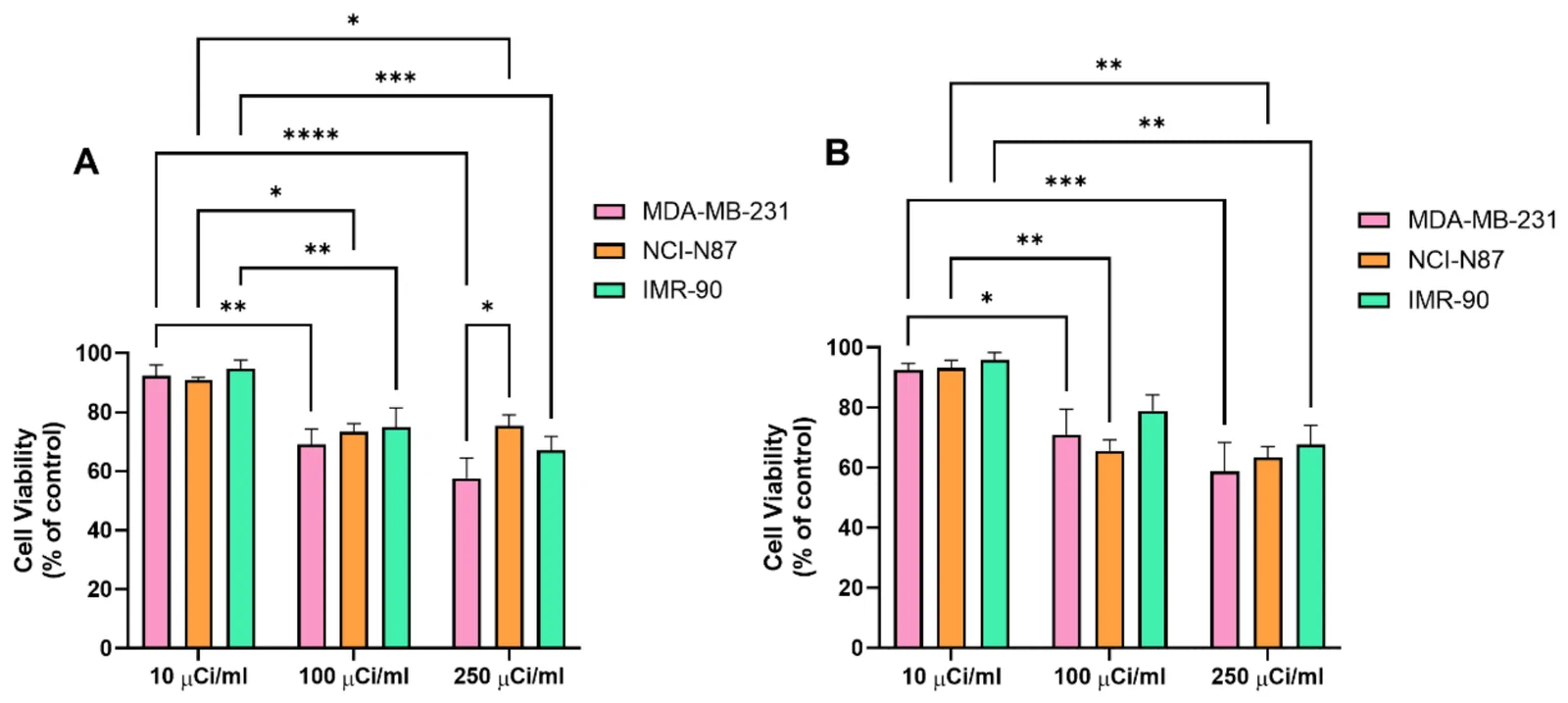
XTT-derived metabolic activity after [^64^Cu]CuCl_2_ treatment. MDA-MB-231, NCI-N87, and IMR-90 metabolic activity after [^64^Cu]CuCl_2_ treatment at three different concentrations for 96 h (**A**) and 120 h (**B**) was measured using CyQUANT™ XTT Assay. Data are shown as the mean ± SEM of five independent experiments and were analyzed by two-way ANOVA with Tukey’s multiple comparison test. * *p* < 0.05; ** *p* < 0.01; *** *p* < 0.001; **** *p* < 0.0001.

Metabolic activity was assessed after 96 h and 120 h of exposure to [^64^Cu]CuCl_2_ at three concentrations (10 μCi/mL, 100 μCi/mL, and 250 μCi/mL) in MDA-MB-231, NCI-N87, and IMR-90 cell lines (Figure 3).

After 96 h, both the 100 μCi/mL and 250 μCi/mL concentrations resulted in a significantly greater reduction in metabolic activity compared to the 10 μCi/mL concentration. No significant differences were observed between 100 μCi/mL and 250 μCi/mL, suggesting that increasing the dose beyond 100 μCi/mL does not confer additional benefit. Similarly, after 120 h of [^64^Cu]CuCl_2_ exposure in both breast and gastric carcinoma cell lines, the 100 μCi/mL and 250 μCi/mL concentrations resulted in a significantly greater reduction in metabolic activity compared to 10 μCi/mL. In contrast, in the IMR-90 normal fibroblast control cells, a significant reduction in metabolic activity was observed only at the highest concentration (250 μCi/mL) compared to 10 μCi/mL. Consistent with the results at 96 h, no significant difference was observed between 100 μCi/mL and 250 μCi/mL at 120 h.

### 2.4. Cell Dosimetry

Table 1 shows the cell self-absorbed dose to the nucleus and the cytoplasm per unit of cumulated activity in the cell for each of the cell lines studied in this work. The results were compared with the values previously reported by Cai et al. [14], which were calculated using Monte Carlo N-Particle (MCNP). The percentage differences for the self-absorbed dose to the nucleus (SADN) were less than 1.5%. Unfortunately, the authors did not provide data on the self-absorbed dose to the cytoplasm (SADC).

The Table 1 shows how the self-absorbed dose by a single cell from each ^64^Cu disintegration depends on the size of the cell compartments. The NCI-N87 cell line exhibits notably higher SADN and SADC values than the other two cell lines due to its smaller size. Additionally, the scale factor is approximately inversely proportional to the mass ratio of the compartments.

**Figure 4:**
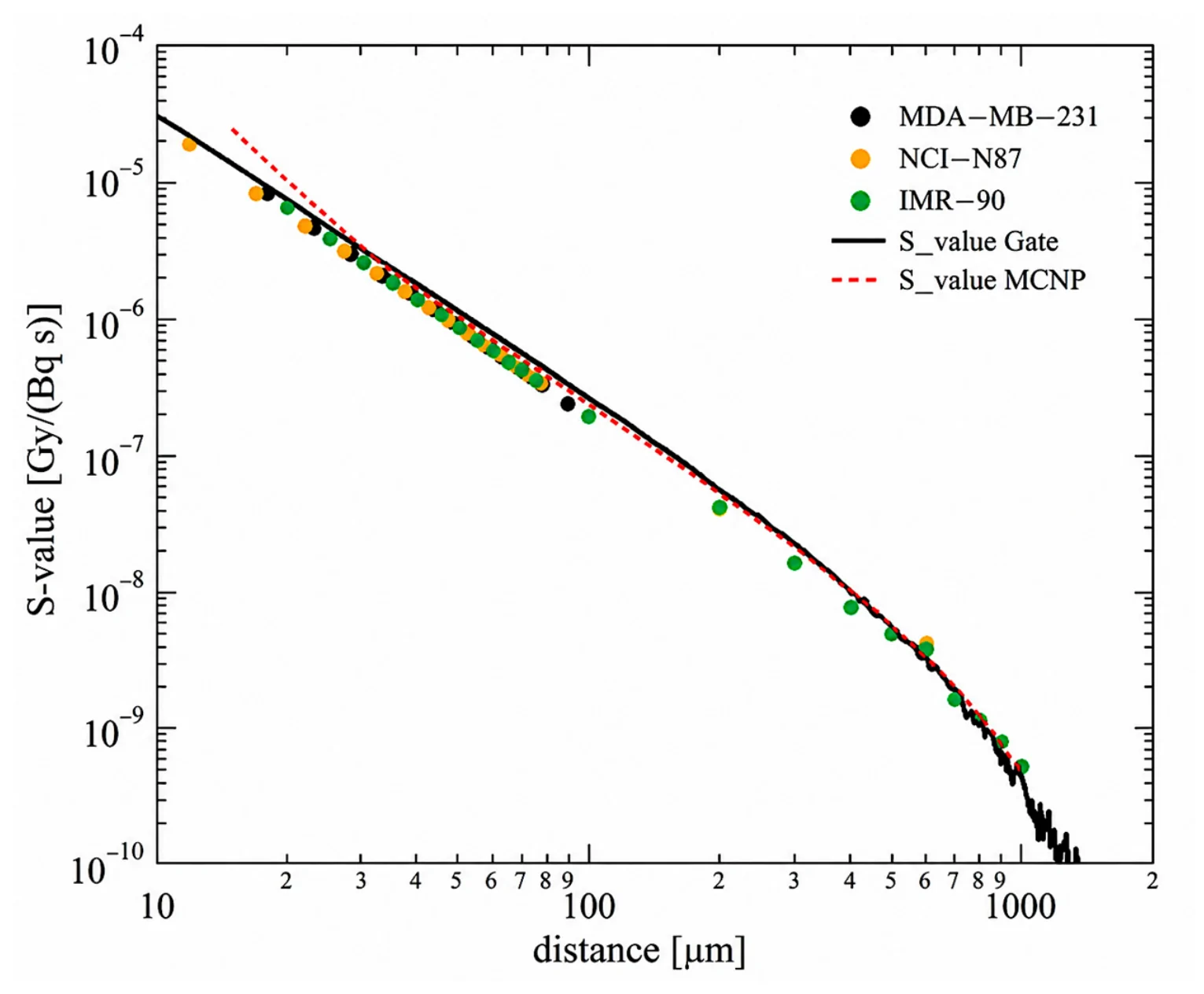
S-values calculated using a pair-cell model for all the three cell lines as function of the distance between source and target cells. In addition, we have plotted the S-value calculated by using the Monte Carlo Gate and the value published by Tse et al. [16] using Penelope Code.

Figure 4 illustrates the average absorbed dose to the second cell per unit of cumulative activity in the first cell, as calculated by the MIRDCell pair-cell model, as a function of the distance between the cells. Each value is the weighted average of the absorbed dose to the nucleus and cytoplasm of the second cell. These values are equivalent to the S-value of ^64^Cu in water when the source is within the first cell and the entire second cell serves as the target. Additionally, we have included the S-values calculated by us with Gate and by Tse et al. [15,16] with the Penelope code in the same figure for comparison purposes.

The results of the MIRD Cell 2D model after 96 h of incubation with [^64^Cu]CuCl_2_ are reported in Table 2 and Table 3 for apoptosis and metabolic activity assays, respectively. The data are presented as a function of labeling percentage for all cell lines. Instead, the results for the 120-h exposure of the overall cell lines are reported in Appendix A (Table A1 and Table A2).

The estimated dose delivered to the three cell lines from the non-internalized radioactivity present in the well after 96 h of incubation, as determined by GATE, is 0.26 ± 0.03 mGy, 2.7 ± 0.3 mGy, and 6.75 ± 0.8 mGy, respectively, for activity concentrations of 10 uCi/mL, 100 uCi/mL, and 250 uCi/mL. These values are at least 300 times lower than the average values calculated for the lowest labeling fraction of each cell line. Therefore, we considered them negligible compared to the internalized contribution.

As expected, the estimated average dose absorbed by a single cell is proportional to its line’s uptake and labeling fraction. Dose values range from a few tenths to a few hundred Gy. Once again, the smaller cell line receives a higher dose for the same labeling fraction, even though the internalized activity per cell is approximately half that of the MDA-MB-231 line. A comparison of the three cell lines reveals that the IMR-90 healthy control line receives the lowest dose in all experiments.

A comparison of Table 2 and Table 3 indicates that the average dose per cell is higher in metabolic activity assays than in apoptosis assays. This is because the average distance between cells is smaller in metabolic activity assays. Consequently, the dose imparted by neighboring cells is greater in metabolic activity assays (see Figure 4).

**Figure A1:**
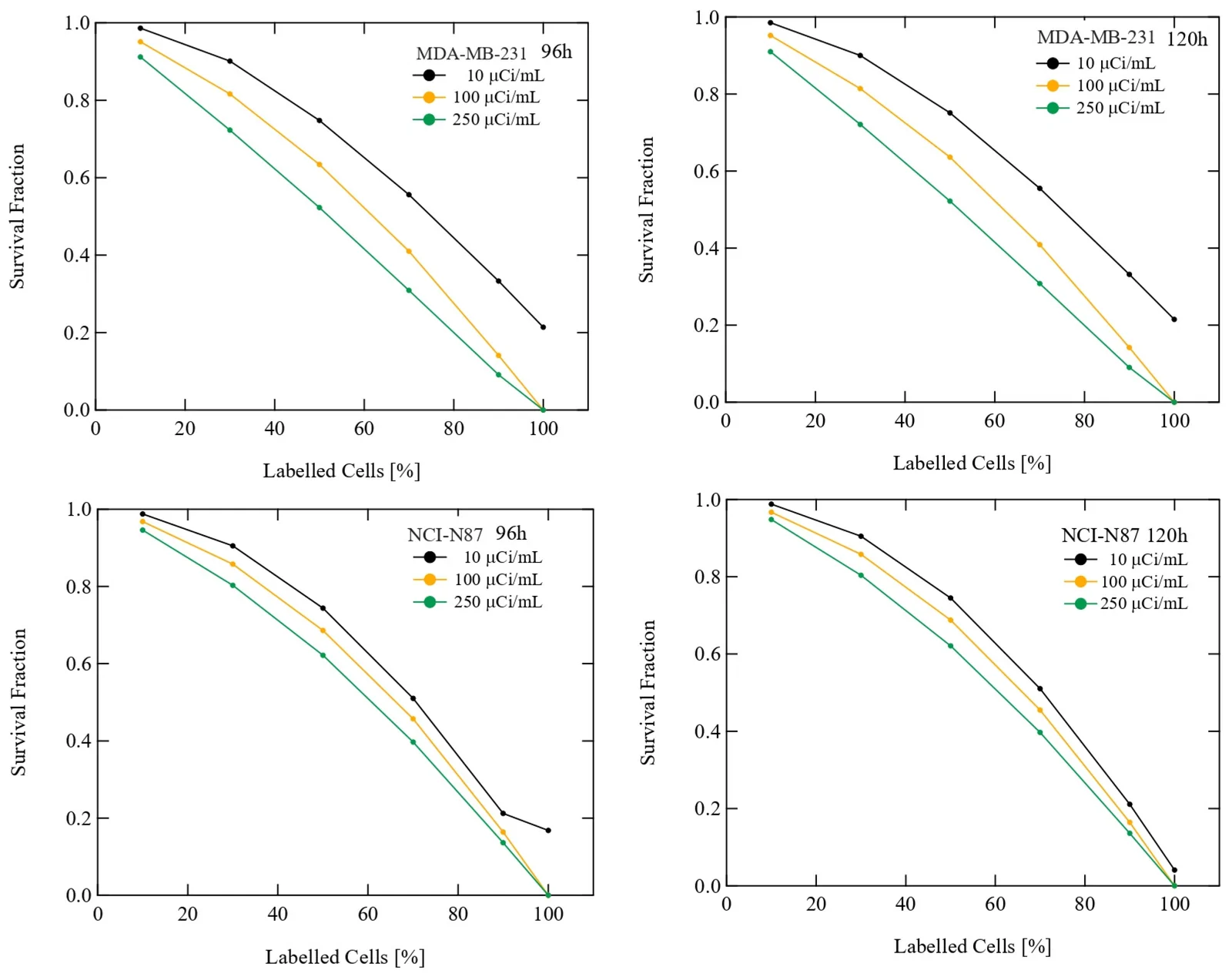

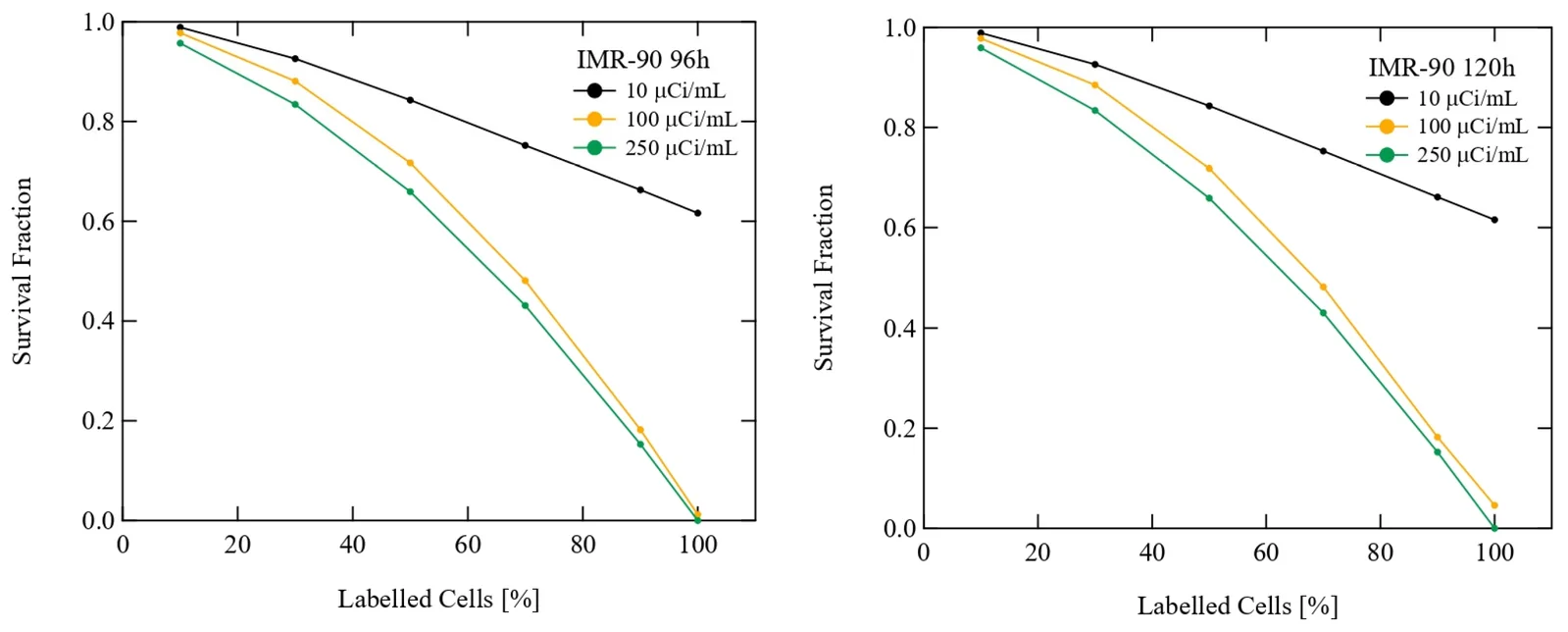
The surviving fraction was calculated as a function of the percentage of labelled cells for the three cell lines after 96 and 120 h from the start of treatment in the apoptosis assay (see left and right plots, respectively). The labelled fractions used in the calculations were 10%, 30%, 50%, 70%, 90% and 100%.

**Figure A2:**
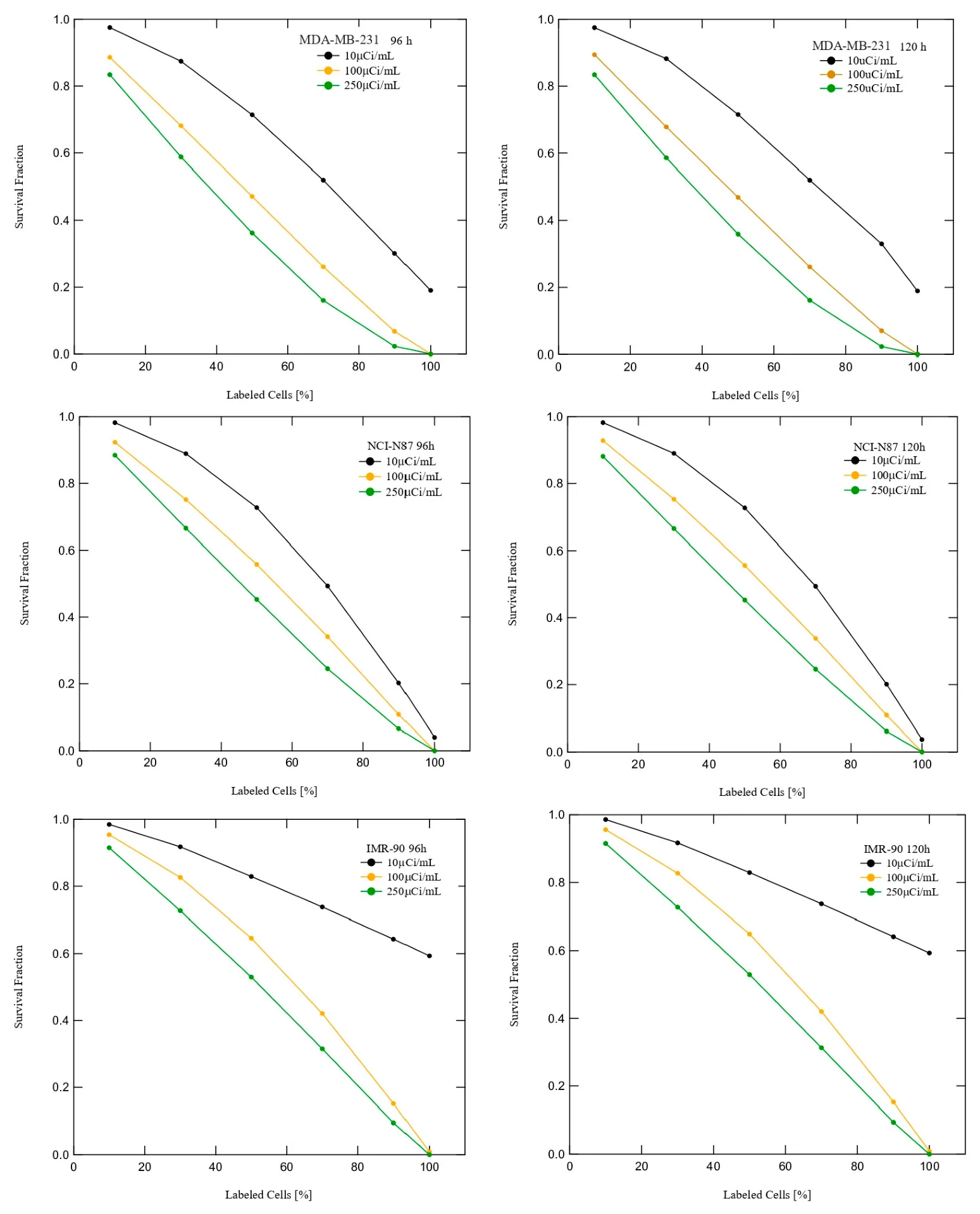
The surviving fraction was calculated as a function of the percentage of labelled cells for the three cell lines after 96 and 120 h from the start of treatment in the XTT-derived metabolic activity assay (see left and right plots, respectively). The labelled fractions used in the calculations were 10%, 30%, 50%, 70%, 90% and 100%.

Figure A1 and Figure A2 in Appendix A plot the corresponding surviving fractions, calculated for both apoptosis and metabolic activity assays, as a function of the percentage of labelled cells (%lab) for each cell line for 120-h incubation times. Regarding the survival curves, it is interesting to note that, for all cell lines, the fraction of cells that survive is approximately (100 −%lab) for the highest activity concentrations of 100 μCi/mL and 250 μCi/mL. This suggests that cells that internalize the ^64^Cu, at the average activities measured in this study, do not survive. At the lowest concentration of 10 μCi/mL, the IMR-90 cell line exhibits a higher survival rate than the other two cell lines.

## 3. Discussion

The objective of this study was to characterize the cellular uptake, subcellular distribution, and biological effects of the radioisotope ^64^Cu, administered as [^64^Cu]CuCl_2_, in a panel of cancer and noncancer cell lines under controlled in vitro conditions. Among the clinical settings that could benefit from such an approach, peritoneal carcinomatosis remains an unresolved challenge in current oncological practice.

The unique radio-physical properties of ^64^Cu, including its decay via β^−^ emission, β^+^ emission, or electron capture, make it particularly interesting as a theranostic radiopharmaceutical. The therapeutic efficacy of ^64^Cu is closely linked to its nuclear decay characteristics. While β^−^ particles have sufficient tissue penetration to affect hundreds of tumor cells, Auger electrons, with their lower energy but significantly higher LET, are particularly effective against single metastatic cells and smaller metastases when precisely targeted to the cell nucleus. This is a crucial advantage, especially given the formidable challenge of tumor genetic instability, which often limits the efficacy of receptor-targeted and antigen-based therapies. By leveraging the selective uptake of Cu^2+^ ions and their nuclear localization, [^64^Cu]CuCl_2_ offers a promising strategy to bypass these limitations. Furthermore, because Auger electrons exert their cytotoxic action, noted for their low penetration power, it is fundamental that the radionuclide producing them reaches the proximity of DNA to cause damage. Unlike conventional targeted radionuclides, free ionic [^64^Cu]CuCl_2_ bypasses the need for exogenous vectors by exploiting endogenous copper transport mechanisms. This feature accounts for the distinct intracellular trafficking and compartmentalized accumulation observed in our cancer cell models.

To investigate the cellular radiobiological effects of [^64^Cu]CuCl_2_, we employed two carcinoma cell lines spanning distinct tumor phenotypes. NCI-N87 was selected because it is a well-established gastric epithelial carcinoma line and is directly relevant to gastric cancer, one of the major primary tumors associated with peritoneal dissemination. MDA-MB-231 was selected as a complementary model of an aggressive, poorly differentiated, highly invasive carcinoma, allowing us to test whether the uptake and radiobiological effects of ionic ^64^Cu extend beyond a single tumor context. Despite originating from breast tissue, MDA-MB-231 is a useful model for studying aggressive tumor behavior and dissemination-related traits in preclinical cancer research [17,18,19]. Although IMR-90 fibroblasts do not reproduce epithelial-specific copper handling, they provide a well-characterized non-malignant human cellular model for assessing whether the uptake and cytotoxic effects of [^64^Cu]CuCl_2_ differ between transformed and non-transformed cells.

Our results demonstrate a significant difference between malignant and healthy cells in the ability to internalize the radioisotope and the resulting biological response. In particular, the breast carcinoma cell line MDA-MB-231 showed markedly higher intracellular uptake than not only IMR-90 fibroblasts but also compared with the gastric carcinoma line NCI-N87. This uptake is radioactivity-dependent and accompanied by an increased localization of the radioisotope for MDA-MB-231 also in the nuclear compartment, suggesting an ability of the more aggressive tumor cells to internalize and retain [^64^Cu]CuCl_2_, likely due to altered copper metabolism.

In contrast with our findings, Serban et al. [20] reported similar incorporation of ^64^Cu ions in colon carcinoma cell lines (HT29 and HCT116), prostate carcinoma cells (DU145), and normal human BJ fibroblasts, with no clear difference related to malignant versus non-malignant origin. This apparent discrepancy suggests that ^64^Cu uptake is unlikely to represent a universal marker distinguishing malignant from non-malignant cells. Instead, uptake and retention may vary substantially among cellular models, depending on the regulation of copper transport, intracellular accumulation, trafficking, and efflux. A direct comparison between the two studies is also limited by the lack of a common reference cell line, such as BJ fibroblasts or HT29 cells. Thus, the preferential uptake observed here in MDA-MB-231 cells, and to a lesser extent in NCI-N87 cells, should be regarded as specific to the experimental panel analyzed in this study. Likewise, the biological effects of [^64^Cu]CuCl_2_ exposure may reflect not only the amount of intracellular radioactivity, but also intrinsic differences in DNA damage response, apoptotic sensitivity, and tolerance to radiation-induced metabolic stress.

The greater difference in the effect of [^64^Cu]CuCl_2_ on tumor cells was reflected to an even greater extent in the apoptotic response. MDA-MB-231 showed the highest level of apoptosis, with a plateau effect already at 100 μCi/mL. Although less pronounced than in MDA, the apoptotic response observed in NCI-N87 cells was still substantial, indicating a sensitivity of this tumor line to [^64^Cu]CuCl_2_ exposure as well. In contrast, apoptosis in normal fibroblasts remained limited even at the highest concentrations, supporting a preferential induction of apoptosis in tumor cells. Analysis of XTT-derived metabolic activity after exposure to [^64^Cu]CuCl_2_ showed less pronounced differences between tumor and non-tumor cells than those observed in the apoptosis assay. This apparent discrepancy should be interpreted in light of the specific biological meaning of the XTT readout. The assay measures the ability of metabolically active cells to reduce tetrazolium salt to formazan and therefore provides an indirect estimate of residual cellular metabolic activity, rather than a direct measurement of clonogenic survival or irreversible cell death. Accordingly, metabolic activity may persist, at least transiently, in cells that are already committed to apoptosis. This is particularly relevant for MDA-MB-231 cells, in which apoptosis was already evident at concentrations as low as 10 μCi/mL, whereas the reduction in XTT-derived metabolic activity at the same time points and activity concentration was only moderate. The combination of these findings suggests that Annexin V/SYTOX staining is a more sensitive indicator of early radiation-induced apoptotic commitment, whereas the XTT assay provides complementary information on treatment-induced metabolic impairment.

Dosimetric calculations confirmed that, at the highest activity concentrations (100 μCi/mL and 250 μCi/mL), the absorbed dose delivered to the cells reaches levels incompatible with cell survival. These findings indicate that cells internalizing ^64^Cu at the average activity levels measured in this study are unlikely to survive, supporting the hypothesis of a significant cytotoxic effect associated with intracellular ^64^Cu accumulation.

While we recognize that peritoneal carcinomatosis inherently develops as complex, three-dimensional (3D) avascular nodules, the utilization of a standardized two-dimensional (2D) monolayer culture was a deliberate and essential approach in this study. This setup was instrumental in establishing a baseline, high-resolution radiobiological characterization of intrinsic cellular uptake, microdosimetry, and early apoptotic responses at the single-cell level, eliminating confounding variables related to mass transport. Building upon these foundational 2D insights, future investigations employing 3D multicellular tumor spheroids will provide a valuable platform to further evaluate penetration kinetics and refine these dosimetric projections under more complex geometrical conditions. Moreover, in a living system, complex pharmacokinetic barriers, physiological copper homeostatic regulation, competing tissue distribution, and macro-vascular tumor microenvironments will heavily govern the agent’s behavior. Therefore, before any conclusions on clinical translatability can be drawn, dedicated in vivo preclinical animal studies remain strictly mandatory to comprehensively evaluate these systemic parameters.

Although [^64^Cu]CuCl_2_ has radiobiological features of interest, several limitations need to be acknowledged. In particular, the absence of a specific molecular target, together with suboptimal biodistribution and physiological accumulation in the liver and kidneys, may reduce therapeutic selectivity and increase the risk of off-target toxicity [21,22]. Furthermore, tumor uptake is often heterogeneous, likely reflecting differences in the expression and activity of copper transporters across tumor types and within the same lesion [23].

To overcome these limitations, different strategies may be envisaged. Locoregional administration could reduce systemic exposure and limit non-target accumulation [22]. In addition, the development of targeted delivery systems may improve tumor retention and biodistribution [21]. Finally, altered copper metabolism in cancer cells may influence intracellular accumulation, although whether this can translate into therapeutic efficacy requires dedicated in vivo validation [23].

## 4. Materials and Methods

Radioactive copper [^64^Cu]CuCl_2_ was produced by A.C.O.M. (Advanced Center Oncology Macerata S.r.l., Macerata, Italy). NE-PER Nuclear and Cytoplasmic Extraction Reagents, CyQUANT XTT Assay, Alexa Fluor 488-labeled Annexin V, SYTOX^®^ AADvanced™ Dead Cell Stain Kit, Annexin Binding Buffer (5X), and Attune NxT Flow Cytometer were purchased from Thermo Fisher Scientific (Waltham, MA, USA). Dulbecco’s modified Eagle’s medium–low glucose (DMEM), fetal bovine serum (FBS), penicillin–streptomycin, phosphate-buffered saline (PBS), and trypsin-EDTA were purchased from Sigma-Aldrich, Merck KGaA (Darmstadt, Germany).

### 4.1. Cell Lines

Human tumor cell lines (MDA-MB-231, human breast adenocarcinoma cell line; NCI-N87, human gastric carcinoma cell line) and a non-transformed reference cell line (IMR-90, normal human fetal lung fibroblast cell line) were obtained from the American Type Culture Collection (ATCC, Manassas, VA, USA). These cells were cultured in DMEM–low glucose, with 10% (*v*/*v*) FBS and 1% penicillin–streptomycin solution, and maintained at 37 °C in a humidified incubator with 5% CO_2_.

### 4.2. [^64^Cu]CuCl_2_ Cellular Uptake Assay

[^64^Cu]CuCl_2_ cellular uptake was assessed with NE-PER Nuclear and Cytoplasmic Extraction Reagents (Thermo Fisher Scientific) that allow the separation and preparation of cytoplasmic and nuclear extracts from mammalian cultured cells [24]. MDA-MB-231, NCI-N87, and IMR-90 seeded in 6-well plates at a density of 8 × 10^5^ cells per well were incubated for 4 h with different concentrations of [^64^Cu]CuCl_2_, 10 µCi/mL, 100 µCi/mL, and 250 µCi/mL. After incubation, the culture medium was collected into a scintillation vial. The cells were quickly washed with 1 mL of PBS, and the wash solution was then transferred into the same vial before measuring radioactivity. The cells were detached with 300 µL of trypsin and then washed with 500 µL of PBS. The suspension was transferred into an Eppendorf tube and centrifuged at 500× *g* for 5 min, RT. After centrifugation, the supernatant was discarded, and 200 µL of ice-cold Cytoplasmic Extraction Reagent I (CER I) was added to the pellet. The sample was vortexed vigorously for 15 s and incubated on ice for 10 min. Subsequently, 11 µL of ice-cold Cytoplasmic Extraction Reagent II (CER II) was added, followed by vortexing for 5 s. The sample was then incubated on ice for an additional 1 min. After incubation, the sample was vortexed for 5 s and centrifuged at 14,000× *g* for 5 min, RT. The supernatant was carefully collected into scintillation vials for subsequent measurement of cytoplasmic radioactivity. The pellet was lysed with 200 µL of 10% Triton-X and transferred to scintillation vials for nuclear radioactivity counting. Radioactivity in each fraction was counted in a Tri-Carb 2810 TR liquid scintillation analyzer (Perkin Elmer, Waltham, MA, USA).

### 4.3. Apoptosis Assay

MDA-MB-231, NCI-N87, and IMR-90 cells were seeded in 24-well plates at a density of 4 × 10^4^ cells per well. Apoptosis was measured after 96 or 120 h of treatment with increasing concentrations of [^64^Cu]CuCl_2_ (10, 100, and 250 µCi/mL) by flow cytometry analysis. After the incubation time, culture medium was collected, cells were detached with trypsin, and samples were double-stained with 30 µL of Annexin V Alexa Fluor™ 488 Ready Flow Conjugate (Thermo Fisher Scientific) and 900 µL of SYTOX^®^ AADvanced™ Dead Cell Stain Kit (Thermo Fisher Scientific), both diluted in Annexin Binding Buffer [25]. After a 10-min incubation at RT, the samples were analyzed using the Attune NxT Flow Cytometer (Thermo Fisher Scientific) equipped with a 488 nm excitation laser.

### 4.4. In Vitro Cytotoxicity Assay

MDA-MB-231, NCI-N87, and IMR-90 cells were seeded in 96-well plates at a density of 2 × 10^4^ cells per well and incubated for 96 or 120 h with increasing concentrations of [^64^Cu]CuCl_2_ (10, 100, and 250 µCi/mL). Metabolic activity was assessed with CyQUANT XTT Assay (Thermo Fisher Scientific) based on the metabolic reduction of the water-soluble yellow tetrazolium salt XTT (2,3-bis-(2-methoxy-4-nitro-5-sulfophenyl)-2H-tetrazolium-5-carboxanilide) into a water-soluble orange formazan dye by viable, metabolically active cells [26]. Following incubation, 6 mL of XTT reagent was combined with 1 mL of electron-coupling reagent to prepare the working solution, and then 70 µL of the working solution was added to each well containing 100 µL of fresh cell culture medium. Metabolic activity was assessed after a 4-h incubation period by measuring absorbance at 450 nm and 660 nm using the EnSight Multimode Plate Reader (Perkin Elmer). Absorbance specific to the XTT assay is measured at 450 nm, with a reference wavelength of 660 nm used to subtract background absorbance due to cell debris or other non-specific components.

### 4.5. Cell Dosimetry

The dosimetric modeling used in this work can be summarized as follows:

*-Cell geometrical model*. The cell is delimited by two concentric spheres, one with a radius R_N_ for the nucleus and one with the radius R_C_ for the outer sphere, delimit the cell. The internal space between the nucleus and the outer spherical surface contains cytoplasm. The cell density is equal to 1 g/cm^3^. It is assumed that the radioactivity within the cell is distributed uniformly between the nucleus and the cytoplasm, despite differing percentages.

*-Multicellular 2D geometry*. The cell population inside a well is arranged on a plane at a specified distance between the cells. The well is circular in shape, and the colony occupies the same area as the well. The distribution of activity among the cell population is uniform.

*-Self and cross-dose calculation*. The absorbed dose of a target cell (*D_t_*) can be obtained by adding together the contributions from the activity in the target cell itself (*D_self_*), the activity in the other cells (*D_cross_*), and the activity in the culture medium (*D_m_*). According to the MIRD scheme, each of these components can be calculated as the product of the S-value corresponding to the relevant source/target combination and the cumulative activity in the source:

(1) $$D_{t}\;=\;D_{self}\;+\;D_{cross}\;+\;D_{m}\;=\;{\tilde{A}}_{t}S_{t\leftarrow t}\;+\;\sum_{s\neq t}{\tilde{A}}_{s}S_{t\leftarrow s}(d_{s})\;+\;{\tilde{A}}_{m}S_{t\leftarrow m}$$

In Equation (1), ${\tilde{A}}_{t}$ , ${\tilde{A}}_{s}$ and ${\tilde{A}}_{m}$ respectively indicate the cumulative activity in the target cell, in the s-th source cell and in the culture medium, while $S_{t\leftarrow t}$ , $S_{t\leftarrow s}$ and $S_{t\leftarrow m}$ are the S values for the coupled target cell/target cell, target cell/source cell and target cell/medium combinations, respectively.

*-Hypothesis on the radioactivity distributions in the cells*.

Based on the results obtained for the MDA-MB-231 and U-87 cell lines by Kim et al. [27] we assumed that the copper concentration within the cell lines would reach a plateau within 2–4 h of incubation with [^64^Cu]CuCl_2_. This would occur if the uptake and washout process of copper in and out of the cell were linear with first-order efflux, as described by the following equation:

(2) $$\frac{dC_{i}}{dt}\;=\;k_{in}C_{ext}\;-\;k_{out}C_{i}$$

In Equation (2), *C_i_*, *C_ext_* indicate the copper concentration inside the cell and in the culture medium, respectively, whereas k_in_ and k_out_ are the uptake and efflux constant, respectively. A possible solution to this differential equation is

(3) $$C_{i}\left(t\right)=\frac{k_{in}}{k_{out}}C_{ext}\left(1-e^{-k_{out}t}\right)$$

**Figure A3:**
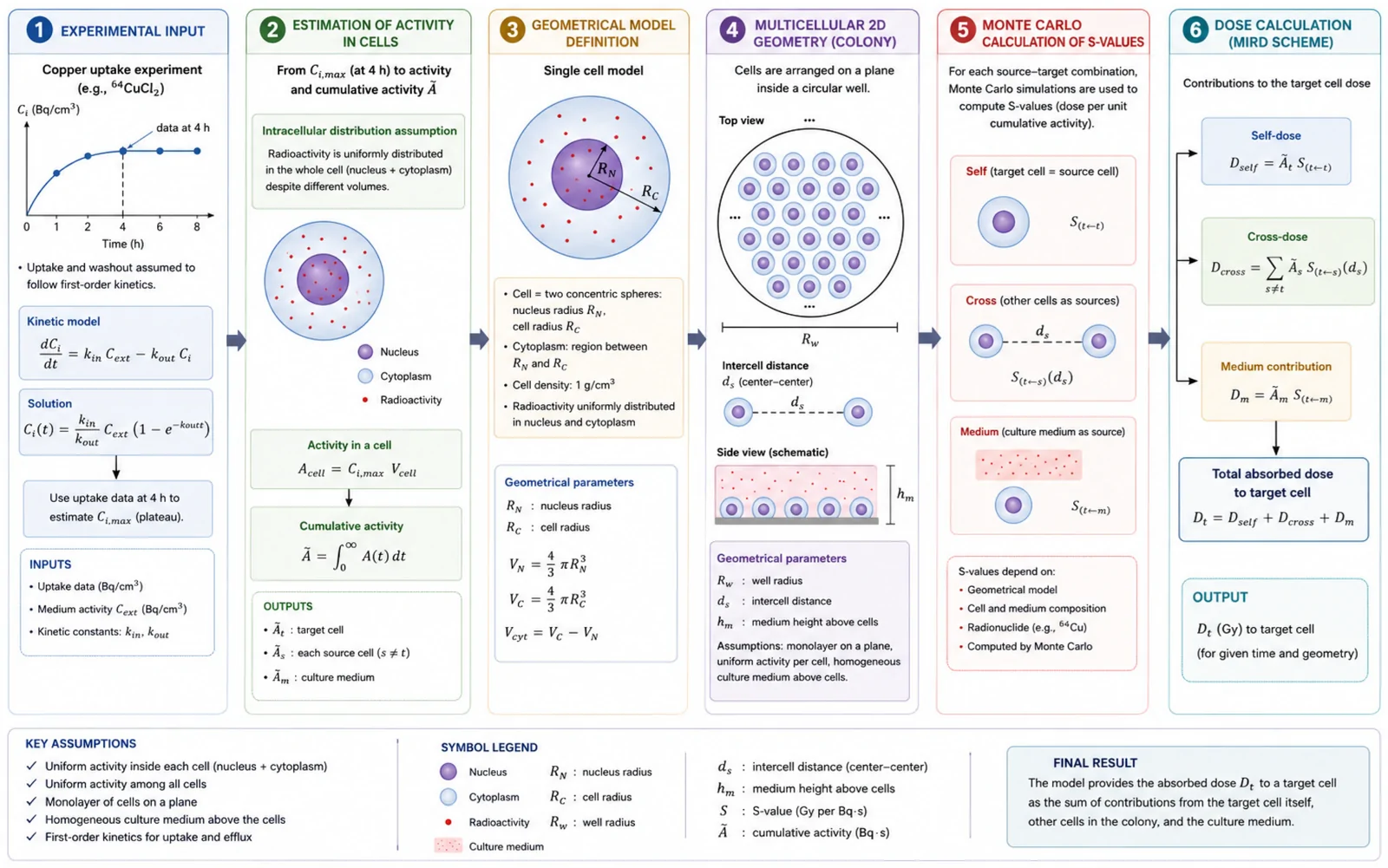
Dosimetric modelling process: from input data to absorbed dose. Workflow of the dosimetric modelling approach used in this work.

So, the uptake data at 4 h were therefore used to calculate the maximum activity in the cell and consequently the cumulative activity (Figure A3).

The dosimetric calculation was performed using MIRDCell v3.13 software [28,29]. The software can calculate the radiation absorbed dose and the survival responses for a single cell, cell pairs, and a 2-dimensional (2-D) and 3-dimensional (3-D) cell populations. The cell radii used in the present study are reported in Table 4. Additionally, GATE v10 Monte Carlo [24] has been used to estimate the average dose imparted to the cells by the radioactivity in the well that was not internalized, as this contribution is not calculated by MIRDCell v3.13 software.

The 1-D cell pair model was used to assess the self-dose and cross-dose to the nucleus and cytoplasm as a function of distance between cells. In the present study, the distance between the cells was varied from the minimum permitted distance (the outer cell diameter) to 1 mm. The self-dose and cross-dose were calculated for each cell’s position. Just for comparison, the S-value curve for ^64^Cu in water has been calculated using Gate-10, a Monte Carlo code for medical physics simulation built on Geant4 code [15]. Instead, the dose assessment, both in apoptosis and metabolic activity assays, has been performed using the MIRDCell 2-D model, which enables the creation of a cell population residing on a plane. In this case, the cell packing density can be adjusted by changing the distance between the cells. In our case, the distance was set so that the number of cells in a well plate is equal to the seeded cells. The mean activity for the cells was obtained from the uptake study, as was the percentage distribution of activity between the nucleus and the cytoplasm for each of the three cell lines. The surviving fraction was calculated as a function of the percentage of labelled cells (%lab = 10, 30, 50, 70, 90, and 100%) for each cell line and activity concentration used in the XTT assay. The distribution of activity among the labelled cells is controlled by %lab specified by the user. All activity distributions undergo a normalization within MIRDcell to satisfy the following mathematical relationship:

(4) $$A_{lab}=\frac{\langle A\rangle }{\%lab}$$

To model the surviving fraction of a cell population based on the absorbed dose, we have used a modified linear-quadratic model, in which the probability that a cell survives is described by the function:

(5) $$P\left(r_{k}\right)=e^{-\alpha_{self}\cdot D_{self}}\times e^{-\alpha_{cross}\cdot D_{cross}}$$

with a_self_ = a_cross_ = 0.51 ± 0.005 Gy^−1^, as recommended by Cal et al. [14]. The time-integrated activity coefficients for the XTT assay were calculated on the assumption that the activity accumulated in each cell decays exponentially at a rate equal to the decay time of ^64^Cu. This assumption is justified by the fact that the cells reach maximum uptake within two hours of being incubated with the radioactivity [26,31].

### 4.6. Statistical Analysis

Data are expressed as mean ± SEM of five independent experiments. All statistical analyses were performed with GraphPad Prism 10.4.2. The differences between groups were analyzed using two-way ANOVA followed by Tukey’s multiple comparisons test. A *p*-value < 0.05 was considered statistically significant.

## 5. Conclusions

In conclusion, this study establishes a rigorous in vitro radiobiological and microdosimetric framework for ionic [^64^Cu]CuCl_2_. By demonstrating a clear, dose-dependent nuclear internalization and selective apoptotic response in specific cancer cell models compared to non-malignant cells, these results provide necessary foundational parameters. However, these in vitro insights do not imply therapeutic readiness. Instead, they serve as a benchmark dataset and a biophysical rationale to justify and optimize the design of future in vivo evaluation.

## Figures and Tables

**Table 1:** The self-absorbed dose to the nucleus (SADN) and cytoplasm (SADC) of a single cell per ^64^Cu decay is calculated using the probability of radionuclide distribution in the nucleus and cytoplasm, which is derived from the uptake data.

Cell Line	SADN [Gy/Bq]	SADC [Gy/Bq]
MDA-MB-231	2.21 × 10^−4^	1.68 × 10^−4^
NCI-N87	1.10 × 10^−3^	4.39 × 10^−4^
IMR-90	2.49 × 10^−4^	1.21 × 10^−4^

**Table 2:** The cell absorbed dose for apoptosis assays with incubation time of 96 h for all cell lines as function of labelling percentage. Absorbed dose values represent theoretical mathematical projections over 96 h calculated by applying a linear first-order efflux kinetic model to the experimental cellular uptake data measured at the 4 h incubation timepoint.

Apoptosis—96 h			
**MDA-MB-231**	**10 µCi/mL**	**100 µCi/mL**	**250 µCi/mL**
**L** **abelled [%]**	**Avg. Dose [Gy]**	**Avg. Dose [Gy]**	**Avg. Dose [Gy]**
10	0.295 ± 0.006	3.85 ± 0.06	11.7 ± 0.2
30	0.90 ± 0.01	11.40 ± 0.06	35.5 ± 0.3
50	1.52 ± 0.01	19.1 ± 0.0	59.4 ± 0.3
70	2.11 ± 0.01	26.83 ± 0.06	82.9 ± 0.2
90	2.71 ± 0.01	34.5 ± 0.01	106.0 ± 0.01
100	3.01 ± 0.01	38.3 ± 0.01	118.0 ± 0.01
**NCI-N87**			
**labelled [%]**	**Avg. Dose [Gy]**	**Avg. Dose [Gy]**	**Avg. Dose [Gy]**
10	0.61 ± 0.04	7.2 ± 0.2	17.3 ± 1.0
30	1.88 ± 0.02	21.5 ± 0.3	52.0 ± 0.7
50	3.11 ± 0.03	35.9 ± 0.2	86.3 ± 0.2
70	4.34 ± 0.02	50.1 ± 0.1	121.0 ± 0.01
90	5.59 ± 0.01	64.4 ± 0.01	156.0 ± 0.01
100	6.21 ± 0.01	71.6 ± 0.01	173.0 ± 0.01
**IMR-90**			
**labelled [%]**	**Avg. Dose [Gy]**	**Avg. Dose [Gy]**	**Avg. Dose [Gy]**
10	0.091 ± 0.003	0.87 ± 0.08	2.82 ± 0.06
30	0.284 ± 0.003	2.62 ± 0.03	8.1 ± 0.1
50	0.474 ± 0.002	4.35 ± 0.0	13.4 ± 0.01
70	0.666 ± 0.002	6.10 ± 0.01	18.8 ± 0.01
90	0.856 ± 0.001	7.84 ± 0.01	24.2 ± 0.01
100	0.950 ± 0.001	8.70 ± 0.01	26.8 ± 0.01

**Table 3:** The cell average absorbed dose for metabolic activity assays with incubation time of 96 h for all cell lines as function of labelling percentage. Absorbed dose values represent theoretical mathematical projections over 96 h, calculated by applying a linear first-order efflux kinetic model to the experimental cellular uptake data measured at the 4 h incubation timepoint.

Metabolic Activity—96 h			
**MDA-MB-231**	**10 µCi/mL**	**100 µCi/mL**	**250 µCi/mL**
**L** **abelled [%]**	**Avg. Dose [Gy]**	**Avg. Dose [Gy]**	**Avg. Dose [Gy]**
10	0.33 ± 0.01	4.1 ± 0.2	13.2 ± 0.7
30	0.99 ± 0.01	12.5 ± 0.2	38.3 ± 0.1
50	1.64 ± 0.01	20.8 ± 0.1	63.9 ± 0.7
70	2.29 ± 0.01	29.2 ± 0.1	90.1 ± 0.1
90	2.94 ± 0.01	37.4 ± 0.1	116.0 ± 0.1
100	3.26 ± 0.01	41.5 ± 0.1	128.0 ± 0.1
**NCI-N87**			
**labelled [%]**	**Avg. Dose [Gy]**	**Avg. Dose [Gy]**	**Avg. Dose [Gy]**
10	0.63 ± 0.06	7.3 ± 0.1	17.6 ± 0.2
30	1.91 ± 0.02	22.2 ± 0.4	52.5 ± 0.3
50	3.17 ± 0.04	36.8 ± 0.2	89.1 ± 1.0
70	4.45 ± 0.02	51.3 ± 0.2	123.7 ± 0.6
90	5.71 ± 0.01	65.9 ± 0.1	159.3 ± 0.6
100	6.35 ± 0.01	73.1 ± 0.1	177.0 ± 0.1
**IMR-90**			
**labelled [%]**	**Avg. Dose [Gy]**	**Avg. Dose [Gy]**	**Avg. Dose [Gy]**
10	0.105 ± 0.004	0.98 ± 0.08	2.8 ± 0.1
30	0.305 ± 0.002	2.79 ± 0.08	8.7 ± 0.1
50	0.511 ± 0.006	4.72 ± 0.03	14.4 ± 0.1
70	0.716 ± 0.002	6.53 ± 0.04	20.1 ± 0.1
90	0.917 ± 0.001	8.40 ± 0.01	25.9 ± 0.1
100	1.020 ± 0.001	9.31 ± 0.01	26.7 ± 0.1

**Table 4:** The radii of the nucleus and outer sphere for the cell lines used in the present study.

Cell Line	R_N_ [μm]	R_C_ [μm]	Reference
MDA-MB-231	5	9	[30]
NCI-N87	3	6	[31]
IMR-90	5	10	[32]

## Data Availability

The original contributions presented in this study are included in the article. Further inquiries can be directed to the corresponding author.

## Abbreviations

The following abbreviations are used in this manuscript:

EMA	European Medicines Agency
FDA	Food and Drug Administration
GC	Gastric Cancer
hCTR1	human Copper Transporter-1
HER2	Growth factor Receptor 2
LET	Linear Energy Transfer
MCNP	Monte Carlo N-Particle
PC	Peritoneal Carcinomatosis
PET	Positron Emission Tomography
PM	Peritoneal Metastasis
LDRIT	Radioimmunotherapy Linear dichroism
SADC	Self-Absorbed Dose to the Cytoplasm
SADN	Self-Absorbed Dose to the Nucleus

## Appendix A

**Table A1:** The cell absorbed doses for apoptosis assays with incubation time of 120 h for all cell lines as function of labelling percentage. Absorbed dose values represent theoretical mathematical projections over or 120 h, calculated by applying a linear first-order efflux kinetic model to the experimental cellular uptake data measured at the 4 h incubation timepoint.

Apoptosis—120 h			
**MDA-MB-231**	**10 µCi/mL**	**100 µCi/mL**	**250 µCi/mL**
**L** **abelled [%]**	**Avg. Dose [Gy]**	**Avg. Dose [Gy]**	**Avg. Dose [Gy]**
10	0.318 ± 0.005	3.817 ± 0.17	12.367 ± 0.416
30	0.920 ± 0.015	11.500 ± 0.1	36.167 ± 0.569
50	1.510 ± 0.0	19.133 ± 0.058	59.167 ± 0.153
70	2.113 ± 0.006	26.900 ± 0.0	83.133 ± 0.252
90	2.720 ± 0.0	34.600 ± 0.0	107.000 ± 0.0
100	3.020 ± 0.0	38.400 ± 0.0	119.000 ± 0.0
**NCI-N87**			
**labelled [%]**	**Avg. Dose [Gy]**	**Avg. Dose [Gy]**	**Avg. Dose [Gy]**
10	0.618 ± 0.033	7.513 ± 0.316	16.6 ± 0.0
30	1.863 ± 0.015	21.600 ± 0.3	51.733 ± 0.503
50	3.110 ± 0.010	35.867 ± 0.208	86.560 ± 0.164
70	4.360 ± 0.010	50.367 ± 0.115	121.667 ± 0.577
90	5.613 ± 0.006	64.700 ± 0.0	156.000 ± 0.0
100	6.240 ± 0.0	71.900 ± 0.0	174.000 ± 0.0
**IMR-90**			
**labelled [%]**	**Avg. Dose [Gy]**	**Avg. Dose [Gy]**	**Avg. Dose [Gy]**
10	0.101 ± 0.004	0.91 ± 0.02	2.8 ± 0.3
30	0.308 ± 0.003	2.83 ± 0.06	8.65 ± 0.01
50	0.514 ± 0.008	4.703 ± 0.006	14.43 ± 0.06
70	0.719 ± 0.002	6.56 ± 0.03	20.27 ± 0.06
90	0.921 ± 0.001	8.433 ± 0.006	26.0 ± 0.0
100	1.02 ± 0.0	9.35 ± 0.0	28.8 ± 0.0

**Table A2:** The cell absorbed dose for metabolic activity assays with incubation time of 120 h for all cell lines as function of labelling percentage. Absorbed dose values represent theoretical mathematical projections over or 120 h, calculated by applying a linear first-order efflux kinetic model to the experimental cellular uptake data measured at the 4 h incubation timepoint.

Metabolic Activity—120 h			
**MDA-MB-231**	**10 µCi/mL**	**100 µCi/mL**	**250 µCi/mL**
**L** **abelled [%]**	**Avg. Dose [Gy]**	**Avg. Dose [Gy]**	**Avg. Dose [Gy]**
10	0.34 ± 0.04	4.0 ± 0.1	12.867 ± 0.7
30	1.0 ± 0.03	12.6 ± 0.3	38.267 ± 0.2
50	1.65 ± 0.01	21.0 ± 0.2	64.400 ± 0.3
70	2.31 ± 0.02	29.3 ± 0.0	90.300 ± 0.1
90	2.96 ± 0.0	37.6 ± 0.0	116.000 ± 0.0
100	3.26 ± 0.03	41.6 ± 0.0	129.000 ± 0.0
**NCI-N87**			
**labelled [%]**	**Avg. Dose [Gy]**	**Avg. Dose [Gy]**	**Avg. Dose [Gy]**
10	0.61 ± 0.02	6.5 ± 0.3	17.9 ± 0.3
30	1.91 ± 0.01	22.1 ± 0.5	53.3 ± 1.2
50	3.19 ± 0.03	36.9 ± 0.3	88.3 ± 0.4
70	4.46 ± 0.01	51.6 ± 0.2	124.0 ± 0.0
90	5.74 ± 0.01	66.1 ± 0.1	160.0 ± 0.0
100	6.37 ± 0.0	73.4 ± 0.0	178.0 ± 0.0
**IMR-90**			
**labelled [%]**	**Avg. Dose [Gy]**	**Avg. Dose [Gy]**	**Avg. Dose [Gy]**
10	0.10 ± 0.01	0.91 ± 0.02	2.8 ± 0.2
30	0.38 ± 0.01	2.83 ± 0.06	8.65 ± 0.01
50	0.51 ± 0.01	4.70 ± 0.01	14.43 ± 0.06
70	0.72 ± 0.01	6.56 ± 0.03	20.27 ± 0.06
90	0.92 ± 0.01	8.43 ± 0.01	26.0 ± 0.1
100	1.02 ± 0.01	9.35 ± 0.01	28.8 ± 0.1
